# Crystal structure of a novel one-dimensional zigzag chain-like cobalt(II) coordination polymer constructed from 4,4′-bi­pyridine and 2-hy­droxy­benzoate ligands

**DOI:** 10.1107/S2056989020009482

**Published:** 2020-07-17

**Authors:** Thawanrat Saelim, Kittipong Chainok, Filip Kielar, Nanthawat Wannarit

**Affiliations:** a Department of Chemistry, Faculty of Science and Technology, Thammasat University, Klong Luang, Pathum Thani 12121, Thailand; b Thammasat University Research Unit in Multifunctional Crystalline Materials and Applications (TU-MCMA), Faculty of Science and Technology, Thammasat University, Klong Luang, Pathum Thani 12121, Thailand; c Department of Chemistry, Faculty of Science, Naresuan University, Phitsanulok 65000, Thailand

**Keywords:** cobalt(II), one-dimensional, zigzag, 4,4′-bi­pyridine, 2-hy­droxy­benzoate, crystal structure

## Abstract

A novel one-dimensional zigzag chain-like Co^II^ coordination polymer constructed from 4,4′-bi­pyridine (4,4′-bpy) and 2-hy­droxy­benzoate (2-OHbenz) ligands, namely, *catena*-poly[[(4,4′-bi­pyridine-κ*N*)­(μ-2-hy­droxy­benzoato-κ^2^ *O*:*O*′)(2-hy­droxy­benzoato-κ^2^ *O*,*O*′)cobalt(II)]-μ-4,4′-bi­pyridine-κ^2^ *N*:*N*′-[aquahemi(μ-4,4′-bi­pyridine-κ^2^ *N*:*N*′)(2-hy­droxy­benzoato-κ*O*(2-hy­droxy­benzoato-κ^2^ *O*:*O*′)cobalt(II)], [Co_2_(C_7_H_5_O_3_)_4_(C_10_H_8_N_2_)_2.5_(H_2_O)]_*n*_, has been synthesized by reacting cobalt(II) nitrate trihydrate, 4,4′-bpy and 2-hy­droxy­benzoic acid in a mixture of water and methanol at room temperature. There are two independent Co^II^ centers, Co1 and Co2, in the asymmetric unit, revealing a distorted octa­hedral geometry with chromophore types of [CoN_2_O_4_] and [CoN_2_O_3_O′], respectively. The Co1 ions are doubly bridged by 2-OHbenz ligands with *syn*–*anti* coordination mode, generating a dinuclear unit. The bridging 4,4′-bpy ligands connect these dinuclear units and the mononuclear Co2 chromophores, providing a one-dimensional alternating zigzag chain-like structure. In the crystal, inter­molecular hydrogen bonds, C—H⋯π and π–π stacking inter­actions are observed and these help to consolidate the packing. In addition, the physical properties of the title compound are reported.

## Abstract 2

The synthesis, crystal structure and physical properties of a novel one-dimensional zigzag chain-like Co^II^ coordination polymer, [Co_2_(2-OHbenz)_4_(4,4′-bpy)_2.5_(H_2_O)]_*n*_, constructed from 4,4′-bi­pyridine (4,4′-bpy) and 2-hy­droxy­benzoate (2-OHbenz) are reported.

## Chemical context   

The design and construction of new coordination polymers (CPs) is of current inter­est and attracts researchers in the fields of modern structural chemistry and materials science because of their potential applications in areas such as ion-exchange, catalysis, sensors, magnetism, and non-linear optics (Dzhardimalieva & Uflyand, 2017 ▸; Loukopoulos & Kostakis, 2018 ▸; Horike *et al.*, 2020 ▸). It is well known that the construction of CPs depends on a variety of factors such as the nature of metal ions and the organic ligands, the molar ratio of the reactants, and the reaction conditions *e.g*. reaction time, pH, solvents, and temperature (Kitagawa *et al.*, 2004 ▸; Noro *et al.*, 2009 ▸). The structure–property relationships of hybrid polymeric materials with 4,4′-bi­pyridine (4,4′-bipy) have been studied intensively (Biradha *et al.*, 2006 ▸; Khrizanforova *et al.*, 2020 ▸). This is because 4,4′-bpy is a rigid mol­ecule that can link the metal centers to form a network with well-defined structures and also support the stability of the structures through aromatic π–π and C—H⋯π inter­actions (Kaes *et al.*, 2000 ▸). Furthermore, many researchers incorporate carboxyl­ate-based ligands for the construction of CPs, giving rise to frameworks with a variety of dimensions and topologies (Gu *et al.*, 2019 ▸; Horike *et al.*, 2020 ▸). Benzoate and its derivatives have been widely used to construct the CPs because of the variety of their coordination modes, resulting in a variety of coordination geometries for the metal centers and inter­esting properties and applications of their CPs (Tong *et al.*, 2000 ▸; Busskamp *et al.*, 2007 ▸; Zhang *et al.*, 2007 ▸; Song *et al.*, 2009 ▸).
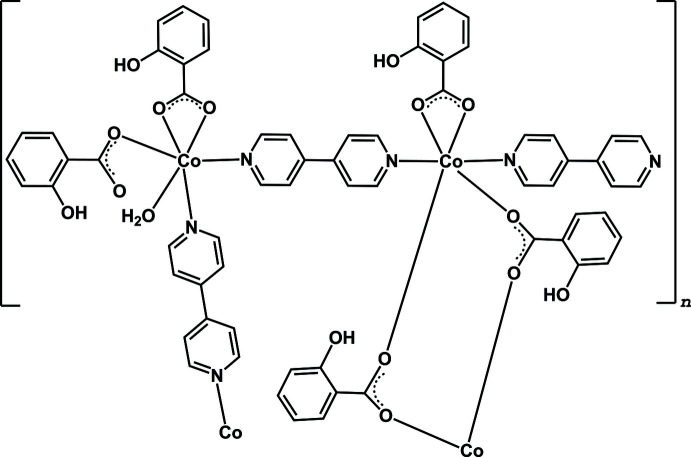


This work was undertaken as part of a search for new first-row transition-metal coordination polymers constructed from 4,4′-bpy and carboxyl­ate ligands. The Co^II^ ion and hy­droxy­benzoate derivatives such as 2-hy­droxy­benzoate (2-OHbenz), 3-hy­droxy­benzoate (3-OHbenz) and 4-hy­droxy­benzoate (4-OHbenz) have been utilized for this. As a result, a Co^II^ coordination polymer containing 4,4′-bpy and 2-OHbenz, [Co_2_(2-OHbenz)_4_(4,4′-bpy)_2.5_(H_2_O)]_*n*_, with a novel 1D alternating zigzag chain-like structure has been successfully synthesized and characterized and its crystal structure has been determined. Herein, we report the synthesis and crystal structure and physical properties of this compound.

## Structural commentary   

**Figure 1:**
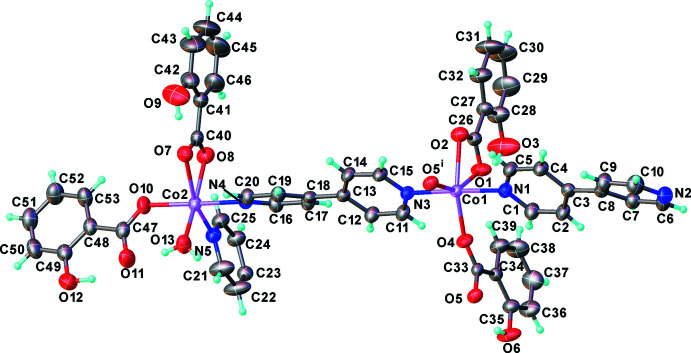
A segment of [Co_2_(2-OHbenz)_4_(4,4′-bpy)_2.5_(H_2_O)]_*n*_ with the atom-labeling scheme. Displacement ellipsoids are drawn at the 50% probability level. [Symmetry code: (i) −*x* + 1, −*y*, −*z* + 1.]

**Figure 2:**
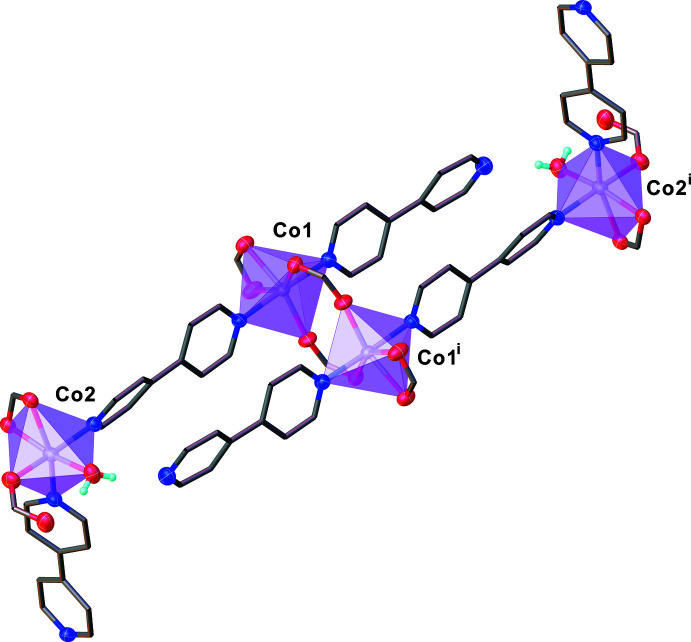
The coordination environments of Co^II^ centers in the title compound. The hydrogen atoms on the aromatic rings and phenol ring on 2-OHbenz ligands have been omitted for clarity. [Symmetry code: (i) −*x* + 1, −*y*, −*z* + 1.]

**Figure 3:**
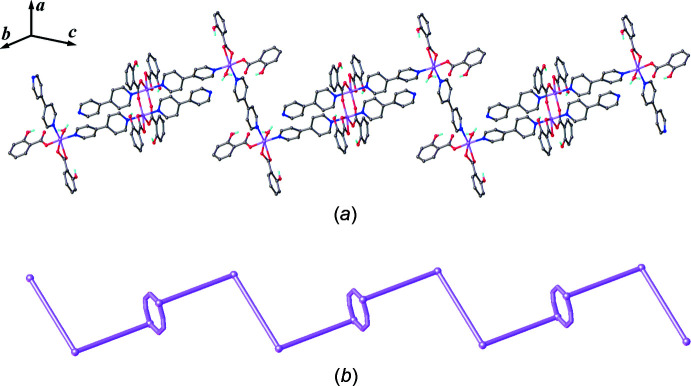
(*a*) View of the one-dimensional alternating zigzag chain-like structure and (*b*) the schematic skeleton representing the topology of the title compound

The asymmetric unit consists of two independent Co^II^ atoms, two and a half of 4,4′-bpy ligands, four 2-OHbenz ligands and one water mol­ecule (Fig. 1 ▸). Both Co^II^ centers exhibit a distorted octa­hedral geometry with [CoN_2_O_4_] and [CoN_2_O_3_O′] chromophores for Co1 and Co2, respectively (Fig. 2 ▸). The Co1 ion is coordinated by two N atoms from two 4,4′-bpy ligands with different monodentate and bridging coordination modes in a *trans*-configuration, and four O atoms from carboxyl­ate groups of one terminal chelating and two bridging 2-OHbenz ligands, while the Co2 ion is bound to two N atoms of two 4,4′-bpy linkers in a *cis*-configuration, and four O atoms from carboxyl­ate groups of two terminally monodentate and chelating 2-OHbenz ligands, and an aqua ligand. The Co—O and Co—N bond lengths fall in the ranges 2.0408 (14)–2.348 (15) and 2.1177 (16)–2.1568 (17) Å, respectively. Two Co1 centers are doubly bridged by two bridging 2-OHbenz ligands with a *syn*–*anti* coordination mode to form a discrete dinuclear unit. The dinuclear units are connected to Co2 atoms by the bridging 4,4′-bpy ligands, providing a one-dimensional zigzag chain-like structure along [101] (Fig. 3 ▸). The Co1⋯Co1^i^ [symmetry code: (i) = −*x* + 1, −*y*, −*z* + 1] and Co1⋯Co2 distances are 4.099 (2) and 11.381 (2) Å, respectively.

**Figure 4:**
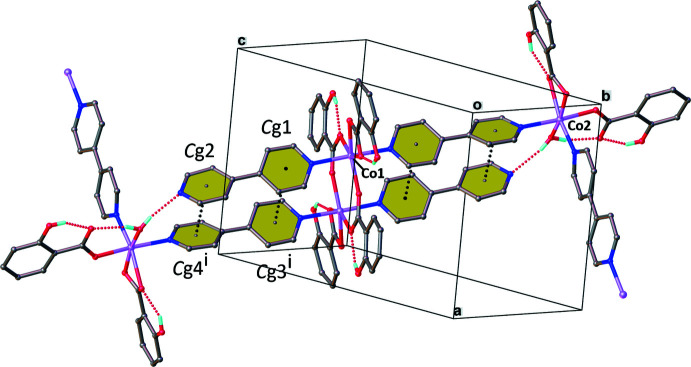
The intra­molecular inter­actions in the title compound. The hydrogen atoms on aromatic rings have been omitted for clarity. *C*g1, *C*g2, *C*g3 and *C*g4 are the centroids of N1/C1–C5, N2/C6–C10, N3/C11–C15 and N4/C16–C20 rings, respectively. [Symmetry code: (i) −*x* + 1, −*y*, −*z* + 1.]

Intra­molecular hydrogen bonds (Table 1 ▸) comprise (i) O—H⋯O inter­actions formed by hydrogen donor atoms from the hydroxyl groups of 2-OHbenz and aqua ligands to oxygen acceptors in the carboxyl­ate groups of the 2-OHbenz ligands and (ii) an O—H⋯N inter­action formed by a hydrogen-atom donor of the aqua ligand to an uncoordinated nitro­gen acceptor atom in the terminal 4,4′-bpy ligand. The intra­molecular π–π stacking inter­actions involve the pyridyl rings of the 4,4′-bpy ligands, the inter­centroid distances *Cg*1⋯*Cg*3^i^ and *Cg*2⋯*Cg*4^i^ being 3.965 (1) and 3.652 (1) Å, respectively, where *Cg*1, *Cg*2, *Cg*3 and *Cg*4 are the centroids of the N1/C1–C5, N2/C6–C10, N3/C11–C15 and N4/C16–C20 rings, respectively [symmetry code: (i) −*x* + 1, −*y*, −*z* + 1; Fig. 4 ▸].

## Supra­molecular features   

**Figure 5:**
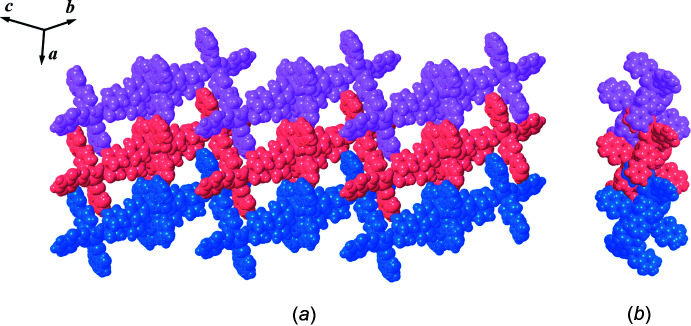
(*a*) Top and (*b*) side views of packing diagram in the (110) plane with a space-filling plot of adjacent one-dimensional zigzag chains of the title compound.

The extended structure of the title compound is consolidated by hydrogen bonds and π–π stacking and C—H⋯π inter­actions. The details of these weak inter­actions are summarized in Tables 1 ▸ and 2 ▸. The inter­molecular inter­actions between the adjacent 1D zigzag chains are (i) C—H⋯O hydrogen bonds between the benzene rings and hydroxyl groups of 2-OHbenz, (ii) π–π stacking inter­actions between the bridging 4,4′-bpy and the terminal chelating 2-OHbenz and also between the phenyl rings of terminal chelating 2-OHbenz ligands, (iii) C—H⋯π inter­actions between the C—H of the terminal monodentate 2-OHbenz ligand and the pyridine ring of 4,4′-bpy ligands and (iv) C—H⋯π inter­actions between the terminal chelating 2-OHbenz ligands. A packing diagram showing adjacent 1D zigzag chains in the (110) plane is shown in Fig. 5 ▸.

## Database survey   

No transition-metal CPs related to the title compound containing a 1D alternating zigzag chain-like structure have been reported. To the best of our knowledge, some related 1D chain-like Co^II^ CPs containing 4,4′-bpy and benzoate or hy­droxy­benzoate derivatives have been reported with two different topologies. The 1D ladder-like structure topology has been found for two Co^II^ CPs, namely, [Co_2_(4,4′-bpy)_3_(H_2_O)_2_(phba)_2_](NO_3_)_2_·4H_2_O, (phba = 4-hy­droxy­benzoate) (MEDROC; Tong *et al.*, 2000 ▸) and [Co_2_(μ_2_-4,4′-bpy)_2_(μ_2_-benz)_2_(benz)_2_]_*n*_, (benz = benzoate) (RIPSUF; Zhang *et al.*, 2007 ▸), while a normal 1D zigzag chain-like structure has been found for [Co_2_(benz)_4_(4,4′-bpy)_2_]_*n*_ (RIPSUF01; Song *et al.*, 2009 ▸).

## Synthesis and crystallization   

A solution of 4,4′-bpy (0.1562 g, 1.0 mmol) in MeOH (5 mL) was slowly added into a solution of Co(NO_3_)_2_·6H_2_O (0.2910 g, 1.0 mmol) in a 4:1 mixture of methanol and water (10 mL). The resulting solution was stirred for 20 min. Next, a solution of 2-OHbenzH (0.1382 g, 1.0 mmol) in methanol (5 mL) was slowly added dropwise and stirred over a period of 15 min. After that, the mixture was filtered. The filtered solution was left to stand without disturbance and allowed to slowly evaporate in the air. After five days, red crystals suitable for single crystal X-ray diffraction were obtained [56.18% yield based on cobalt(II) salt]. Elemental analysis; calculated for C_53_H_42_Co_2_N_5_O_13_: C 59.06, H 4.21, N 6.50%; found: C 59.14, H 3.99, N 6.41%. IR (KBr, ν/cm^−1^): 3087*s*, 1595*s*, 1485*s*, 1460*s*, 1459*s*, 1413*m*, 1389*s*, 1359*s*, 1308*w*, 1252*m*, 1218*m*, 1143*w*, 1068*w*, 1029*w*, 871*w*, 814*s*, 749*s*, 701*w*, 671*w*, 633*w*, 530*w*.

The IR spectrum of the title compound (see Fig. S1 in the supporting information) shows a characteristic broad peak centered at 3087 cm^−1^, which is assigned to OH stretching vibrations of the water mol­ecule and the hydroxyl groups of 2-OHbenz. Strong and sharp peaks at 1595 and 1485 cm^−1^ can be assigned as the asymmetric and symmetric COO^−^ stretching vibrations of the chelating 2-OHbenz ligands, respectively. Peaks in the region of 600–1000 cm^−1^ are assigned to CH bending of the aromatic rings in the ligands (Zhu *et al.*, 2016 ▸).

The solid-state electronic spectrum of the title compound (Fig. S2) shows two broad bands in the visible region with the main peak centered about 515 nm (19.42 kK), which can be assigned to the ν_3_: ^4^ *T*_1*g*_ → ^4^ *T*_1*g*_(*P*) transition. There is a small peak as a shoulder at around 655 nm (15.27 kK), assigned to the ν_2_: ^4^ *T*_1*g*_ → ^4^ *A*_2*g*_ transition and a broad band centered about 1095 nm (13.24 kK), which can be assigned to the ν_1_: ^4^ *T*_1*g*_ → ^4^ *T*_2*g*_ transition. The characteristic bands of this electronic spectrum correspond to a distorted octa­hedral geometry for Co^II^ compounds as confirmed by the X-ray structure (Piromchom *et al.*, 2014 ▸).

The PXRD pattern of the title compound (Fig. S3) was used to check the phase purity of the bulk sample in the solid state. The measured PXRD pattern of the title compound closely matches the simulated pattern generated from the single-crystal X-ray diffraction data, confirming the title compound is pure.

The TGA curve shown in (Fig. S4) demonstrates the thermal stability of the title compound up to 160°C. The first weight-loss step of 27.37% is observed from 160 to 277°C and can be attributed to the loss of coordinated water and two 2-OHbenz mol­ecules. The next step weight-loss step of 25.7% observed from 277 to 356°C corresponds to the loss of a coordinated 2-OHbenz mol­ecule. Finally, the weight loss of about 36.33% from 356 to 520°C can be assigned to the removal of two and half of the 4,4′-bpy ligands. The residual product is assumed to be CoO.

The solid-state photoluminescent properties of the title compound and free ligands were investigated at room temperature. As shown in Fig. S5, the emission spectra of the free ligands 4,4′-bpy and 2-OHbenzH (λ_ex_ = 340 nm) exhibit strong emission bands at 425 and 439 nm, respectively. However, no detectable emission can be observed for the title compound (λ_ex_ = 340 nm). This complete PL quenching is the result of the low energy *d*–*d* transitions in the partially filled metal ion centers found for Co^II^ compounds described above and reported elsewhere (Yang *et al.*, 2012 ▸; Zhu *et al.*, 2014 ▸).

## Refinement   

Crystal data, data collection and structure refinement details are summarized in Table 3 ▸. C-bound H atoms were positioned geometrically and refined using a riding model, with C—H = 0.93 Å and the *U*_iso_(H) = 1.2*U*_eq_(C). O-bound H atoms were located in a difference electron-density map, and were refined with bond-length restraints of O—H = 0.84 (1) Å.

## Supplementary Material

Crystal structure: contains datablock(s) I. DOI: 10.1107/S2056989020009482/is5544sup1.cif

Structure factors: contains datablock(s) I. DOI: 10.1107/S2056989020009482/is5544Isup2.hkl

Supporting information file. DOI: 10.1107/S2056989020009482/is5544sup3.pdf

CCDC reference: 2015747

Additional supporting information:  crystallographic information; 3D view; checkCIF report

## Figures and Tables

**Table 1:** Hydrogen-bond geometry (Å, °) *Cg*4, *Cg*7 and *Cg*9 are the centroids of the N4/C16–C20, C34–C39 and C48–C53 rings, respectively.

*D*—H⋯*A*	*D*—H	H⋯*A*	*D*⋯*A*	*D*—H⋯*A*
O3—H3⋯O1	0.85 (3)	1.77 (3)	2.553 (3)	153 (4)
O6—H8⋯O5	0.84 (2)	1.86 (2)	2.587 (2)	145 (2)
O9—H13⋯O7	0.84 (2)	1.79 (2)	2.568 (3)	152 (4)
O12—H18⋯O11	0.85 (2)	1.74 (2)	2.531 (2)	154 (3)
O13—H26⋯O11	0.84 (1)	1.82 (2)	2.625 (2)	160 (2)
O13—H27⋯N2^i^	0.83 (2)	2.05 (2)	2.860 (3)	167 (2)
C25—H25⋯O8	0.93	2.55	3.143 (3)	122
C39—H39⋯O1	0.93	2.38	3.271 (3)	160
C5—H5⋯O12^ii^	0.93	2.59	3.324 (3)	136
C6—H6⋯O7^iii^	0.93	2.48	3.300 (3)	147
C12—H12⋯O3^iv^	0.93	2.58	3.173 (3)	122
C15—H15⋯O6^v^	0.93	2.51	3.292 (3)	142
C2—H2⋯*Cg*9^iii^	0.93	2.95	3.833 (2)	160
C31—H31⋯*Cg*4^vi^	0.93	2.85	3.681 (4)	149
C51—H51⋯*Cg*7^vii^	0.93	2.72	3.623 (3)	165

Symmetry codes: (i) 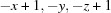; (ii) 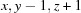; (iii) 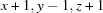; (iv) 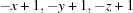; (v) 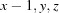; (vi) 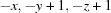; (vii) 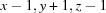.

**Table 2:** Analysis of short ring inter­actions (Å) *Cg*(I) and *Cg*(*J*) are the centroids of rings *I* and *J*; *CgI*_Perp is the perpendicular distance of *Cg*(*I*) on ring *J* and slippage is the distance between *Cg*(*I*) and the perpendicular projection of *Cg*(*J*) on ring *I. *Cg**1, *Cg*2, *Cg*3, *Cg*4, *Cg*5, *Cg*6, *Cg*7 and *Cg*8 are the centroids of the N1/C1–C5, N2/C6–C10, N3/C11–C15, N4/C16–C20, N5/C21–C25, C27–C32, C34–C39 and C41–C46 rings, respectively.

*Cg*(*I*)	*Cg*(*J*)	Symmetry_*Cg*(*J*)	*Cg*(*I*)⋯*Cg*(*J*)	*CgI*_Perp	*CgJ*_Perp	Slippage
*Cg*1	*Cg*3	−*x* + 1, −*y*, −*z* + 1	3.9651 (13)	3.7526 (9)	3.6199 (8)	1.618
*Cg*2	*Cg*4	−*x* + 1, −*y*, −*z* + 1	3.6515 (13)	3.5063 (9)	3.5546 (8)	0.836
*Cg*5	*Cg*8	−*x*, −*y* + 2, −*z*	4.0381 (19)	3.5840 (10)	3.5978 (13)	1.832
*Cg*6	*Cg*7	−*x* + 1, −*y* + 1, −*z* + 1	4.2986 (18)	4.2205 (13)	3.9319 (10)	1.737
*Cg*6	*Cg*8	*x*, −*z* + 1, −*z* + 1	3.814 (2)	3.7674 (13)	3.7372 (14)	0.765

**Table 3:** Experimental details

Crystal data	
Chemical formula	[Co_2_(C_7_H_5_O_3_)_4_(C_10_H_8_N_2_)_2.5_(H_2_O)]
*M*_r_	1074.77
Crystal system, space group	Triclinic, *P* 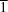
Temperature (K)	296
*a*, *b*, *c* (Å)	10.8832 (18), 11.4742 (19), 19.905 (3)
α, β, γ (°)	74.295 (5), 89.791 (5), 88.502 (6)
*V* (Å^3^)	2392.0 (7)
*Z*	2
Radiation type	Mo *K*α
μ (mm^−1^)	0.77
Crystal size (mm)	0.32 × 0.24 × 0.2

Data collection	
Diffractometer	Bruker D8 Quest CMOS Photon II
Absorption correction	Multi-scan (*SADABS*; Bruker, 2016 ▸)
*T*_min_, *T*_max_	0.677, 0.746
No. of measured, independent and observed [*I* > 2σ(*I*)] reflections	60283, 9771, 7780
*R*_int_	0.042
(sin θ/λ)_max_ (Å^−1^)	0.625

Refinement	
*R*[*F* ^2^ > 2σ(*F* ^2^)], *wR*(*F* ^2^), *S*	0.035, 0.088, 1.05
No. of reflections	9771
No. of parameters	682
No. of restraints	6
H-atom treatment	H atoms treated by a mixture of independent and constrained refinement
Δρ_max_, Δρ_min_ (e Å^−3^)	0.43, −0.30

Computer programs: *APEX3* and *SAINT* (Bruker, 2016 ▸), *SHELXT* (Sheldrick, 2015*a* ▸), *SHELXL* (Sheldrick, 2015*b* ▸) and *OLEX2* (Dolomanov *et al.*, 2009 ▸).

## supplementary crystallographic information

### Crystal data

**Table:**

[Co_2_(C_7_H_5_O_3_)_4_(C_10_H_8_N_2_)_2.5_(H_2_O)]	*Z* = 2
*M**_r_* = 1074.77	*F*(000) = 1106
Triclinic, *P*1	*D*_x_ = 1.492 Mg m^−^^3^
*a* = 10.8832 (18) Å	Mo *K*α radiation, λ = 0.71073 Å
*b* = 11.4742 (19) Å	Cell parameters from 9918 reflections
*c* = 19.905 (3) Å	θ = 3.1–28.2°
α = 74.295 (5)°	µ = 0.77 mm^−^^1^
β = 89.791 (5)°	*T* = 296 K
γ = 88.502 (6)°	Block, red
*V* = 2392.0 (7) Å^3^	0.32 × 0.24 × 0.2 mm

### Data collection

**Table:**

Bruker D8 Quest CMOS Photon II diffractometer	9771 independent reflections
Radiation source: sealed x-ray tube, Mo	7780 reflections with *I* > 2σ(*I*)
Graphite monochromator	*R*_int_ = 0.042
Detector resolution: 7.39 pixels mm^-1^	θ_max_ = 26.4°, θ_min_ = 3.0°
ω and φ scans	*h* = −13→13
Absorption correction: multi-scan (SADABS; Bruker, 2016)	*k* = −14→14
*T*_min_ = 0.677, *T*_max_ = 0.746	*l* = −24→24
60283 measured reflections	

### Refinement

**Table:**

Refinement on *F*^2^	Primary atom site location: dual
Least-squares matrix: full	Hydrogen site location: mixed
*R*[*F*^2^ > 2σ(*F*^2^)] = 0.035	H atoms treated by a mixture of independent and constrained refinement
*wR*(*F*^2^) = 0.088	*w* = 1/[σ^2^(*F*_o_^2^) + (0.0419*P*)^2^ + 0.8816*P*] where *P* = (*F*_o_^2^ + 2*F*_c_^2^)/3
*S* = 1.05	(Δ/σ)_max_ < 0.001
9771 reflections	Δρ_max_ = 0.43 e Å^−^^3^
682 parameters	Δρ_min_ = −0.29 e Å^−^^3^
6 restraints	

### Special details

**Table:**

Geometry. All esds (except the esd in the dihedral angle between two l.s. planes) are estimated using the full covariance matrix. The cell esds are taken into account individually in the estimation of esds in distances, angles and torsion angles; correlations between esds in cell parameters are only used when they are defined by crystal symmetry. An approximate (isotropic) treatment of cell esds is used for estimating esds involving l.s. planes.

### Fractional atomic coordinates and isotropic or equivalent isotropic displacement parameters (Å^2^)

**Table:**

	*x*	*y*	*z*	*U*_iso_*/*U*_eq_	
Co1	0.38320 (2)	0.10363 (2)	0.54244 (2)	0.02725 (7)	
Co2	0.06977 (2)	0.72632 (2)	0.02018 (2)	0.03175 (8)	
O1	0.40605 (13)	0.25879 (14)	0.58404 (9)	0.0484 (4)	
O2	0.23200 (13)	0.16721 (13)	0.59633 (8)	0.0409 (3)	
O3	0.4298 (2)	0.4553 (2)	0.61905 (15)	0.0980 (9)	
H3	0.443 (4)	0.398 (2)	0.6003 (19)	0.121 (14)*	
O4	0.55720 (12)	0.11808 (13)	0.50300 (8)	0.0402 (3)	
O5	0.72507 (12)	0.03072 (12)	0.47519 (7)	0.0344 (3)	
O6	0.93656 (14)	0.12657 (17)	0.47886 (11)	0.0602 (5)	
H8	0.888 (2)	0.077 (2)	0.4705 (14)	0.071 (9)*	
O7	−0.11751 (13)	0.67237 (13)	0.06159 (7)	0.0406 (3)	
O8	−0.03026 (13)	0.83546 (13)	0.07254 (8)	0.0438 (4)	
O9	−0.3306 (2)	0.6158 (2)	0.11428 (13)	0.0875 (7)	
H13	−0.2608 (17)	0.610 (3)	0.0971 (18)	0.106 (13)*	
O10	−0.00286 (14)	0.81713 (14)	−0.07657 (7)	0.0454 (4)	
O11	0.10789 (16)	0.73590 (15)	−0.14789 (8)	0.0543 (4)	
O12	0.08458 (17)	0.81806 (18)	−0.27880 (9)	0.0592 (5)	
H18	0.107 (3)	0.775 (2)	−0.2386 (9)	0.088 (11)*	
O13	0.13809 (16)	0.59112 (14)	−0.02174 (8)	0.0441 (4)	
H26	0.135 (3)	0.623 (2)	−0.0648 (6)	0.078 (10)*	
H27	0.2003 (16)	0.549 (2)	−0.0066 (13)	0.063 (9)*	
N1	0.44634 (15)	−0.01847 (15)	0.63825 (8)	0.0339 (4)	
N2	0.66774 (18)	−0.43591 (18)	0.94863 (9)	0.0474 (5)	
N3	0.32399 (14)	0.22088 (14)	0.44421 (8)	0.0311 (3)	
N4	0.13327 (15)	0.61430 (14)	0.11989 (8)	0.0332 (4)	
N5	0.22979 (15)	0.83133 (15)	0.00504 (9)	0.0357 (4)	
C1	0.56246 (19)	−0.0201 (2)	0.66090 (11)	0.0418 (5)	
H1	0.616518	0.035341	0.634347	0.050*	
C2	0.60535 (19)	−0.0993 (2)	0.72121 (11)	0.0417 (5)	
H2	0.687050	−0.097234	0.734268	0.050*	
C3	0.52771 (18)	−0.18256 (17)	0.76293 (10)	0.0331 (4)	
C4	0.40701 (18)	−0.18020 (19)	0.73970 (11)	0.0393 (5)	
H4	0.350822	−0.233833	0.765656	0.047*	
C5	0.37092 (18)	−0.09847 (19)	0.67830 (11)	0.0393 (5)	
H5	0.289795	−0.098811	0.663857	0.047*	
C6	0.7144 (2)	−0.3259 (2)	0.92367 (11)	0.0451 (5)	
H6	0.780326	−0.305313	0.947374	0.054*	
C7	0.67096 (19)	−0.24166 (19)	0.86514 (11)	0.0386 (5)	
H7	0.707240	−0.166564	0.850264	0.046*	
C8	0.57241 (18)	−0.26897 (18)	0.82815 (10)	0.0342 (4)	
C9	0.5219 (2)	−0.3816 (2)	0.85489 (11)	0.0444 (5)	
H9A	0.454541	−0.403962	0.833014	0.053*	
C10	0.5716 (2)	−0.4605 (2)	0.91394 (12)	0.0514 (6)	
H10	0.535930	−0.535569	0.930657	0.062*	
C11	0.39719 (18)	0.30579 (18)	0.40608 (11)	0.0373 (5)	
H11	0.475641	0.312109	0.422913	0.045*	
C12	0.36219 (18)	0.38385 (18)	0.34346 (11)	0.0367 (5)	
H12	0.416736	0.440794	0.318790	0.044*	
C13	0.24535 (17)	0.37796 (17)	0.31686 (10)	0.0299 (4)	
C14	0.16882 (17)	0.29091 (18)	0.35696 (10)	0.0365 (5)	
H14	0.089433	0.283600	0.341728	0.044*	
C15	0.21083 (17)	0.21556 (19)	0.41926 (10)	0.0366 (5)	
H15	0.158001	0.158196	0.445261	0.044*	
C16	0.20919 (19)	0.65071 (18)	0.16245 (10)	0.0376 (5)	
H16	0.237771	0.729246	0.148137	0.045*	
C17	0.24722 (19)	0.57804 (18)	0.22630 (10)	0.0372 (5)	
H17	0.300167	0.607670	0.253975	0.045*	
C18	0.20611 (16)	0.45990 (17)	0.24934 (10)	0.0301 (4)	
C19	0.12639 (18)	0.42283 (18)	0.20528 (10)	0.0345 (4)	
H19	0.095861	0.344987	0.218331	0.041*	
C20	0.09266 (18)	0.50133 (18)	0.14235 (10)	0.0361 (4)	
H20	0.038814	0.474499	0.113901	0.043*	
C21	0.3388 (2)	0.7903 (2)	−0.01008 (16)	0.0613 (7)	
H21	0.343265	0.715188	−0.019510	0.074*	
C22	0.4454 (2)	0.8530 (2)	−0.01252 (17)	0.0668 (8)	
H22	0.519350	0.819901	−0.023310	0.080*	
C23	0.44329 (18)	0.96475 (18)	0.00095 (11)	0.0361 (4)	
C24	0.32980 (19)	1.00692 (19)	0.01651 (12)	0.0419 (5)	
H24	0.322945	1.081478	0.026449	0.050*	
C25	0.22646 (19)	0.93955 (19)	0.01745 (12)	0.0416 (5)	
H25	0.151031	0.971218	0.027200	0.050*	
C26	0.29583 (18)	0.25023 (18)	0.60563 (10)	0.0342 (4)	
C27	0.24654 (18)	0.33984 (19)	0.64101 (11)	0.0382 (5)	
C28	0.3152 (2)	0.4384 (2)	0.64491 (15)	0.0584 (7)	
C29	0.2655 (3)	0.5232 (3)	0.67603 (19)	0.0848 (10)	
H29	0.309833	0.590962	0.676996	0.102*	
C30	0.1514 (3)	0.5073 (3)	0.7053 (2)	0.0926 (11)	
H30	0.119079	0.563885	0.726726	0.111*	
C31	0.0841 (3)	0.4094 (3)	0.7035 (2)	0.0870 (11)	
H31	0.006936	0.398807	0.724094	0.104*	
C32	0.1313 (2)	0.3262 (2)	0.67084 (14)	0.0585 (7)	
H32	0.084907	0.260227	0.668929	0.070*	
C33	0.67051 (16)	0.11563 (17)	0.49469 (9)	0.0278 (4)	
C34	0.74627 (17)	0.21332 (16)	0.50758 (9)	0.0289 (4)	
C35	0.87402 (18)	0.21315 (18)	0.50003 (11)	0.0368 (5)	
C36	0.9411 (2)	0.3034 (2)	0.51642 (14)	0.0539 (6)	
H36	1.026195	0.303308	0.511912	0.065*	
C37	0.8821 (2)	0.3925 (2)	0.53918 (14)	0.0581 (7)	
H37	0.927865	0.451984	0.550389	0.070*	
C38	0.7565 (2)	0.3953 (2)	0.54571 (15)	0.0600 (7)	
H38	0.717158	0.457127	0.560291	0.072*	
C39	0.6895 (2)	0.3055 (2)	0.53040 (13)	0.0454 (5)	
H39	0.604491	0.306589	0.535436	0.055*	
C40	−0.11856 (18)	0.76483 (18)	0.08531 (10)	0.0351 (4)	
C41	−0.2240 (2)	0.7879 (2)	0.12733 (11)	0.0417 (5)	
C42	−0.3247 (2)	0.7133 (3)	0.13934 (14)	0.0596 (7)	
C43	−0.4237 (3)	0.7394 (4)	0.17739 (19)	0.0996 (12)	
H43	−0.491888	0.690437	0.184663	0.119*	
C44	−0.4210 (4)	0.8365 (5)	0.2040 (2)	0.1126 (15)	
H44	−0.487079	0.852323	0.230315	0.135*	
C45	−0.3231 (4)	0.9112 (4)	0.19291 (19)	0.1003 (12)	
H45	−0.322902	0.977912	0.210964	0.120*	
C46	−0.2246 (3)	0.8869 (3)	0.15467 (14)	0.0654 (7)	
H46	−0.157687	0.937517	0.147131	0.079*	
C47	0.03259 (19)	0.81423 (19)	−0.13648 (11)	0.0389 (5)	
C48	−0.01849 (19)	0.90816 (18)	−0.19807 (10)	0.0369 (5)	
C49	0.0107 (2)	0.9058 (2)	−0.26616 (11)	0.0408 (5)	
C50	−0.0378 (2)	0.9945 (2)	−0.32279 (12)	0.0532 (6)	
H50	−0.018213	0.992567	−0.368002	0.064*	
C51	−0.1141 (3)	1.0846 (2)	−0.31211 (13)	0.0557 (6)	
H51	−0.146484	1.143341	−0.350112	0.067*	
C52	−0.1430 (3)	1.0884 (2)	−0.24526 (13)	0.0585 (7)	
H52	−0.194411	1.150024	−0.238205	0.070*	
C53	−0.0959 (2)	1.0013 (2)	−0.18913 (12)	0.0492 (6)	
H53	−0.116154	1.004531	−0.144223	0.059*	

### Atomic displacement parameters (Å^2^)

**Table:**

	*U*^11^	*U*^22^	*U*^33^	*U*^12^	*U*^13^	*U*^23^
Co1	0.02255 (13)	0.02972 (14)	0.03064 (14)	−0.00244 (10)	−0.00050 (10)	−0.01000 (11)
Co2	0.03023 (15)	0.03340 (15)	0.03044 (14)	−0.00662 (11)	−0.00272 (11)	−0.00606 (11)
O1	0.0320 (8)	0.0574 (10)	0.0669 (10)	−0.0087 (7)	0.0140 (7)	−0.0352 (8)
O2	0.0345 (8)	0.0414 (8)	0.0535 (9)	−0.0060 (6)	0.0036 (7)	−0.0236 (7)
O3	0.0613 (13)	0.0969 (17)	0.171 (2)	−0.0421 (12)	0.0432 (14)	−0.0927 (18)
O4	0.0227 (7)	0.0500 (9)	0.0494 (9)	−0.0074 (6)	0.0033 (6)	−0.0153 (7)
O5	0.0289 (7)	0.0325 (7)	0.0456 (8)	−0.0027 (6)	−0.0020 (6)	−0.0167 (6)
O6	0.0283 (8)	0.0663 (11)	0.1028 (15)	−0.0096 (8)	0.0154 (9)	−0.0514 (11)
O7	0.0401 (8)	0.0434 (8)	0.0424 (8)	−0.0058 (6)	−0.0009 (6)	−0.0182 (7)
O8	0.0373 (8)	0.0409 (8)	0.0534 (9)	−0.0087 (7)	0.0015 (7)	−0.0127 (7)
O9	0.0702 (15)	0.1036 (17)	0.1071 (18)	−0.0508 (13)	0.0321 (13)	−0.0558 (15)
O10	0.0473 (9)	0.0540 (9)	0.0305 (8)	−0.0016 (7)	−0.0051 (7)	−0.0036 (7)
O11	0.0608 (11)	0.0557 (10)	0.0417 (9)	0.0114 (8)	−0.0053 (8)	−0.0064 (8)
O12	0.0549 (11)	0.0758 (13)	0.0468 (10)	0.0011 (9)	0.0071 (9)	−0.0164 (10)
O13	0.0557 (10)	0.0367 (8)	0.0393 (9)	0.0024 (7)	−0.0089 (8)	−0.0092 (7)
N1	0.0301 (9)	0.0372 (9)	0.0343 (9)	−0.0013 (7)	−0.0024 (7)	−0.0094 (7)
N2	0.0479 (11)	0.0517 (12)	0.0379 (10)	0.0049 (9)	−0.0037 (9)	−0.0049 (9)
N3	0.0268 (8)	0.0334 (9)	0.0318 (8)	−0.0035 (7)	−0.0002 (7)	−0.0064 (7)
N4	0.0327 (9)	0.0344 (9)	0.0316 (8)	−0.0037 (7)	−0.0008 (7)	−0.0070 (7)
N5	0.0326 (9)	0.0345 (9)	0.0385 (9)	−0.0076 (7)	0.0018 (7)	−0.0068 (7)
C1	0.0350 (11)	0.0470 (13)	0.0396 (11)	−0.0116 (9)	−0.0019 (9)	−0.0040 (10)
C2	0.0302 (11)	0.0512 (13)	0.0409 (12)	−0.0069 (9)	−0.0067 (9)	−0.0073 (10)
C3	0.0331 (10)	0.0342 (10)	0.0336 (10)	−0.0006 (8)	−0.0004 (8)	−0.0118 (8)
C4	0.0303 (11)	0.0445 (12)	0.0394 (11)	−0.0076 (9)	0.0015 (9)	−0.0044 (9)
C5	0.0275 (10)	0.0487 (13)	0.0388 (11)	−0.0029 (9)	−0.0024 (9)	−0.0067 (10)
C6	0.0419 (12)	0.0562 (14)	0.0381 (12)	0.0028 (10)	−0.0074 (10)	−0.0149 (11)
C7	0.0382 (11)	0.0393 (11)	0.0386 (11)	−0.0034 (9)	−0.0020 (9)	−0.0108 (9)
C8	0.0309 (10)	0.0381 (11)	0.0345 (10)	0.0023 (8)	0.0007 (8)	−0.0115 (9)
C9	0.0421 (12)	0.0433 (12)	0.0436 (12)	−0.0082 (10)	−0.0071 (10)	−0.0037 (10)
C10	0.0566 (15)	0.0435 (13)	0.0474 (13)	−0.0068 (11)	−0.0021 (11)	−0.0002 (11)
C11	0.0298 (10)	0.0392 (11)	0.0411 (11)	−0.0077 (8)	−0.0068 (9)	−0.0072 (9)
C12	0.0310 (10)	0.0360 (11)	0.0396 (11)	−0.0102 (8)	−0.0012 (9)	−0.0034 (9)
C13	0.0275 (10)	0.0308 (10)	0.0324 (10)	−0.0008 (8)	0.0002 (8)	−0.0099 (8)
C14	0.0230 (9)	0.0440 (12)	0.0393 (11)	−0.0046 (8)	−0.0035 (8)	−0.0055 (9)
C15	0.0248 (10)	0.0434 (12)	0.0369 (11)	−0.0058 (8)	0.0024 (8)	−0.0023 (9)
C16	0.0434 (12)	0.0323 (11)	0.0352 (11)	−0.0083 (9)	−0.0019 (9)	−0.0053 (9)
C17	0.0386 (11)	0.0370 (11)	0.0366 (11)	−0.0077 (9)	−0.0060 (9)	−0.0105 (9)
C18	0.0257 (9)	0.0331 (10)	0.0312 (10)	−0.0001 (8)	0.0031 (8)	−0.0083 (8)
C19	0.0350 (11)	0.0317 (10)	0.0358 (10)	−0.0074 (8)	0.0002 (8)	−0.0070 (8)
C20	0.0355 (11)	0.0389 (11)	0.0339 (10)	−0.0087 (9)	−0.0038 (8)	−0.0092 (9)
C21	0.0431 (14)	0.0479 (14)	0.106 (2)	−0.0135 (11)	0.0171 (14)	−0.0420 (15)
C22	0.0359 (13)	0.0581 (16)	0.122 (2)	−0.0137 (11)	0.0255 (14)	−0.0501 (17)
C23	0.0341 (11)	0.0350 (11)	0.0393 (11)	−0.0083 (8)	0.0037 (9)	−0.0094 (9)
C24	0.0360 (11)	0.0338 (11)	0.0580 (14)	−0.0054 (9)	0.0032 (10)	−0.0158 (10)
C25	0.0325 (11)	0.0388 (12)	0.0526 (13)	−0.0029 (9)	0.0028 (10)	−0.0109 (10)
C26	0.0299 (10)	0.0381 (11)	0.0357 (10)	−0.0003 (8)	0.0004 (8)	−0.0121 (9)
C27	0.0330 (11)	0.0404 (12)	0.0462 (12)	−0.0006 (9)	0.0023 (9)	−0.0201 (10)
C28	0.0446 (14)	0.0581 (15)	0.0853 (19)	−0.0104 (11)	0.0096 (13)	−0.0407 (14)
C29	0.078 (2)	0.073 (2)	0.129 (3)	−0.0116 (16)	0.013 (2)	−0.071 (2)
C30	0.078 (2)	0.091 (2)	0.138 (3)	0.0048 (18)	0.023 (2)	−0.081 (2)
C31	0.0585 (18)	0.097 (2)	0.128 (3)	−0.0012 (17)	0.0335 (19)	−0.070 (2)
C32	0.0396 (13)	0.0647 (16)	0.0810 (19)	−0.0055 (12)	0.0138 (13)	−0.0363 (15)
C33	0.0254 (10)	0.0308 (10)	0.0246 (9)	−0.0042 (7)	−0.0008 (7)	−0.0029 (7)
C34	0.0272 (9)	0.0293 (10)	0.0302 (10)	−0.0034 (7)	0.0011 (8)	−0.0079 (8)
C35	0.0286 (10)	0.0380 (11)	0.0457 (12)	−0.0073 (8)	0.0062 (9)	−0.0139 (9)
C36	0.0348 (12)	0.0587 (15)	0.0755 (17)	−0.0201 (11)	0.0094 (12)	−0.0292 (13)
C37	0.0599 (16)	0.0484 (14)	0.0749 (18)	−0.0248 (12)	0.0078 (13)	−0.0301 (13)
C38	0.0595 (16)	0.0454 (14)	0.087 (2)	−0.0055 (12)	0.0106 (14)	−0.0386 (14)
C39	0.0342 (11)	0.0426 (12)	0.0652 (15)	−0.0023 (9)	0.0045 (10)	−0.0242 (11)
C40	0.0346 (11)	0.0376 (11)	0.0318 (10)	−0.0014 (9)	−0.0060 (8)	−0.0071 (9)
C41	0.0383 (12)	0.0491 (13)	0.0380 (11)	0.0010 (10)	−0.0012 (9)	−0.0125 (10)
C42	0.0449 (14)	0.084 (2)	0.0532 (15)	−0.0119 (13)	0.0088 (12)	−0.0232 (14)
C43	0.0530 (19)	0.157 (4)	0.094 (3)	−0.018 (2)	0.0313 (18)	−0.042 (3)
C44	0.087 (3)	0.166 (4)	0.095 (3)	0.029 (3)	0.030 (2)	−0.056 (3)
C45	0.116 (3)	0.112 (3)	0.089 (3)	0.033 (3)	0.010 (2)	−0.058 (2)
C46	0.0744 (19)	0.0665 (18)	0.0634 (17)	0.0101 (14)	−0.0003 (14)	−0.0325 (14)
C47	0.0361 (11)	0.0408 (12)	0.0378 (12)	−0.0121 (9)	−0.0052 (9)	−0.0061 (9)
C48	0.0381 (11)	0.0371 (11)	0.0341 (10)	−0.0133 (9)	−0.0041 (9)	−0.0057 (9)
C49	0.0376 (11)	0.0456 (12)	0.0382 (11)	−0.0123 (9)	0.0011 (9)	−0.0088 (10)
C50	0.0615 (16)	0.0614 (16)	0.0324 (12)	−0.0167 (13)	−0.0008 (11)	−0.0041 (11)
C51	0.0699 (17)	0.0436 (14)	0.0453 (14)	−0.0119 (12)	−0.0150 (12)	0.0034 (11)
C52	0.0750 (18)	0.0418 (13)	0.0565 (15)	0.0047 (12)	−0.0135 (13)	−0.0100 (12)
C53	0.0622 (15)	0.0460 (13)	0.0398 (12)	−0.0014 (11)	−0.0079 (11)	−0.0124 (10)

### Geometric parameters (Å, º)

**Table:**

Co1—O1	2.1772 (15)	C14—H14	0.9300
Co1—O2	2.1769 (14)	C14—C15	1.377 (3)
Co1—O4	2.0408 (14)	C15—H15	0.9300
Co1—O5^i^	2.0686 (13)	C16—H16	0.9300
Co1—N1	2.1412 (16)	C16—C17	1.376 (3)
Co1—N3	2.1421 (16)	C17—H17	0.9300
Co2—O7	2.2348 (15)	C17—C18	1.393 (3)
Co2—O8	2.1135 (15)	C18—C19	1.389 (3)
Co2—O10	2.0739 (14)	C19—H19	0.9300
Co2—O13	2.0717 (16)	C19—C20	1.375 (3)
Co2—N4	2.1568 (16)	C20—H20	0.9300
Co2—N5	2.1177 (16)	C21—H21	0.9300
Co2—C40	2.514 (2)	C21—C22	1.375 (3)
O1—C26	1.270 (2)	C22—H22	0.9300
O2—C26	1.247 (2)	C22—C23	1.379 (3)
O3—H3	0.845 (10)	C23—C23^ii^	1.488 (4)
O3—C28	1.346 (3)	C23—C24	1.379 (3)
O4—C33	1.244 (2)	C24—H24	0.9300
O5—C33	1.275 (2)	C24—C25	1.378 (3)
O6—H8	0.838 (10)	C25—H25	0.9300
O6—C35	1.348 (3)	C26—C27	1.484 (3)
O7—C40	1.272 (2)	C27—C28	1.391 (3)
O8—C40	1.254 (2)	C27—C32	1.380 (3)
O9—H13	0.839 (10)	C28—C29	1.386 (4)
O9—C42	1.346 (3)	C29—H29	0.9300
O10—C47	1.261 (3)	C29—C30	1.365 (4)
O11—C47	1.266 (3)	C30—H30	0.9300
O12—H18	0.853 (10)	C30—C31	1.366 (4)
O12—C49	1.348 (3)	C31—H31	0.9300
O13—H26	0.837 (10)	C31—C32	1.379 (4)
O13—H27	0.832 (10)	C32—H32	0.9300
N1—C1	1.341 (3)	C33—C34	1.486 (3)
N1—C5	1.338 (3)	C34—C35	1.398 (3)
N2—C6	1.338 (3)	C34—C39	1.390 (3)
N2—C10	1.333 (3)	C35—C36	1.392 (3)
N3—C11	1.340 (2)	C36—H36	0.9300
N3—C15	1.339 (2)	C36—C37	1.369 (3)
N4—C16	1.338 (3)	C37—H37	0.9300
N4—C20	1.338 (2)	C37—C38	1.373 (4)
N5—C21	1.329 (3)	C38—H38	0.9300
N5—C25	1.330 (3)	C38—C39	1.378 (3)
C1—H1	0.9300	C39—H39	0.9300
C1—C2	1.370 (3)	C40—C41	1.478 (3)
C2—H2	0.9300	C41—C42	1.388 (3)
C2—C3	1.387 (3)	C41—C46	1.386 (3)
C3—C4	1.391 (3)	C42—C43	1.387 (4)
C3—C8	1.479 (3)	C43—H43	0.9300
C4—H4	0.9300	C43—C44	1.358 (6)
C4—C5	1.375 (3)	C44—H44	0.9300
C5—H5	0.9300	C44—C45	1.364 (6)
C6—H6	0.9300	C45—H45	0.9300
C6—C7	1.373 (3)	C45—C46	1.379 (4)
C7—H7	0.9300	C46—H46	0.9300
C7—C8	1.392 (3)	C47—C48	1.494 (3)
C8—C9	1.385 (3)	C48—C49	1.398 (3)
C9—H9A	0.9300	C48—C53	1.392 (3)
C9—C10	1.377 (3)	C49—C50	1.393 (3)
C10—H10	0.9300	C50—H50	0.9300
C11—H11	0.9300	C50—C51	1.369 (4)
C11—C12	1.372 (3)	C51—H51	0.9300
C12—H12	0.9300	C51—C52	1.378 (4)
C12—C13	1.390 (3)	C52—H52	0.9300
C13—C14	1.391 (3)	C52—C53	1.372 (3)
C13—C18	1.474 (3)	C53—H53	0.9300
			
O2—Co1—O1	59.82 (5)	C18—C17—H17	120.1
O4—Co1—O1	91.69 (6)	C17—C18—C13	121.87 (17)
O4—Co1—O2	151.47 (6)	C19—C18—C13	121.51 (17)
O4—Co1—O5^i^	117.97 (6)	C19—C18—C17	116.62 (17)
O4—Co1—N1	91.32 (6)	C18—C19—H19	120.0
O4—Co1—N3	87.16 (6)	C20—C19—C18	119.94 (18)
O5^i^—Co1—O1	150.29 (5)	C20—C19—H19	120.0
O5^i^—Co1—O2	90.48 (5)	N4—C20—C19	123.42 (18)
O5^i^—Co1—N1	89.68 (6)	N4—C20—H20	118.3
O5^i^—Co1—N3	89.31 (6)	C19—C20—H20	118.3
N1—Co1—O1	91.38 (6)	N5—C21—H21	118.3
N1—Co1—O2	90.90 (6)	N5—C21—C22	123.4 (2)
N1—Co1—N3	177.53 (6)	C22—C21—H21	118.3
N3—Co1—O1	90.61 (6)	C21—C22—H22	119.8
N3—Co1—O2	91.37 (6)	C21—C22—C23	120.4 (2)
O7—Co2—C40	30.36 (6)	C23—C22—H22	119.8
O8—Co2—O7	60.20 (5)	C22—C23—C23^ii^	122.0 (2)
O8—Co2—N4	89.11 (6)	C22—C23—C24	115.94 (19)
O8—Co2—N5	95.55 (6)	C24—C23—C23^ii^	122.1 (2)
O8—Co2—C40	29.87 (6)	C23—C24—H24	119.7
O10—Co2—O7	91.19 (6)	C25—C24—C23	120.6 (2)
O10—Co2—O8	93.29 (6)	C25—C24—H24	119.7
O10—Co2—N4	173.40 (6)	N5—C25—C24	122.9 (2)
O10—Co2—N5	93.17 (6)	N5—C25—H25	118.5
O10—Co2—C40	93.68 (6)	C24—C25—H25	118.5
O13—Co2—O7	107.21 (6)	O1—C26—C27	118.92 (18)
O13—Co2—O8	167.09 (6)	O2—C26—O1	119.20 (18)
O13—Co2—O10	89.66 (6)	O2—C26—C27	121.87 (18)
O13—Co2—N4	86.68 (6)	C28—C27—C26	120.90 (19)
O13—Co2—N5	96.83 (7)	C32—C27—C26	120.2 (2)
O13—Co2—C40	137.40 (7)	C32—C27—C28	118.9 (2)
N4—Co2—O7	84.66 (6)	O3—C28—C27	122.1 (2)
N4—Co2—C40	85.34 (6)	O3—C28—C29	118.0 (2)
N5—Co2—O7	155.58 (6)	C29—C28—C27	119.8 (2)
N5—Co2—N4	92.71 (6)	C28—C29—H29	120.0
N5—Co2—C40	125.28 (7)	C30—C29—C28	119.9 (3)
C26—O1—Co1	90.17 (12)	C30—C29—H29	120.0
C26—O2—Co1	90.79 (12)	C29—C30—H30	119.6
C28—O3—H3	105 (3)	C29—C30—C31	120.9 (3)
C33—O4—Co1	165.49 (14)	C31—C30—H30	119.6
C33—O5—Co1^i^	117.07 (11)	C30—C31—H31	120.2
C35—O6—H8	110 (2)	C30—C31—C32	119.6 (3)
C40—O7—Co2	87.04 (12)	C32—C31—H31	120.2
C40—O8—Co2	93.04 (12)	C27—C32—H32	119.6
C42—O9—H13	104 (3)	C31—C32—C27	120.7 (2)
C47—O10—Co2	129.12 (15)	C31—C32—H32	119.6
C49—O12—H18	105 (2)	O4—C33—O5	121.83 (17)
Co2—O13—H26	103 (2)	O4—C33—C34	120.09 (17)
Co2—O13—H27	123.7 (19)	O5—C33—C34	118.09 (16)
H26—O13—H27	117 (3)	C35—C34—C33	122.02 (17)
C1—N1—Co1	122.34 (13)	C39—C34—C33	119.26 (17)
C5—N1—Co1	121.01 (13)	C39—C34—C35	118.67 (18)
C5—N1—C1	116.64 (17)	O6—C35—C34	122.54 (18)
C10—N2—C6	115.94 (19)	O6—C35—C36	117.85 (19)
C11—N3—Co1	121.43 (13)	C36—C35—C34	119.59 (19)
C15—N3—Co1	121.58 (13)	C35—C36—H36	119.9
C15—N3—C11	116.95 (16)	C37—C36—C35	120.1 (2)
C16—N4—Co2	124.46 (13)	C37—C36—H36	119.9
C16—N4—C20	116.83 (17)	C36—C37—H37	119.5
C20—N4—Co2	118.69 (13)	C36—C37—C38	121.1 (2)
C21—N5—Co2	123.24 (14)	C38—C37—H37	119.5
C21—N5—C25	116.75 (18)	C37—C38—H38	120.4
C25—N5—Co2	119.69 (14)	C37—C38—C39	119.2 (2)
N1—C1—H1	118.4	C39—C38—H38	120.4
N1—C1—C2	123.22 (19)	C34—C39—H39	119.3
C2—C1—H1	118.4	C38—C39—C34	121.3 (2)
C1—C2—H2	119.8	C38—C39—H39	119.3
C1—C2—C3	120.44 (19)	O7—C40—Co2	62.59 (11)
C3—C2—H2	119.8	O7—C40—C41	119.59 (18)
C2—C3—C4	116.31 (18)	O8—C40—Co2	57.09 (11)
C2—C3—C8	121.21 (18)	O8—C40—O7	119.58 (19)
C4—C3—C8	122.48 (18)	O8—C40—C41	120.83 (19)
C3—C4—H4	120.0	C41—C40—Co2	176.31 (14)
C5—C4—C3	119.91 (19)	C42—C41—C40	121.4 (2)
C5—C4—H4	120.0	C46—C41—C40	120.0 (2)
N1—C5—C4	123.47 (18)	C46—C41—C42	118.6 (2)
N1—C5—H5	118.3	O9—C42—C41	121.9 (2)
C4—C5—H5	118.3	O9—C42—C43	118.2 (3)
N2—C6—H6	118.0	C43—C42—C41	120.0 (3)
N2—C6—C7	124.0 (2)	C42—C43—H43	120.0
C7—C6—H6	118.0	C44—C43—C42	120.0 (3)
C6—C7—H7	120.1	C44—C43—H43	120.0
C6—C7—C8	119.8 (2)	C43—C44—H44	119.4
C8—C7—H7	120.1	C43—C44—C45	121.1 (3)
C7—C8—C3	121.04 (18)	C45—C44—H44	119.4
C9—C8—C3	122.60 (18)	C44—C45—H45	120.3
C9—C8—C7	116.33 (19)	C44—C45—C46	119.5 (3)
C8—C9—H9A	120.1	C46—C45—H45	120.3
C10—C9—C8	119.8 (2)	C41—C46—H46	119.6
C10—C9—H9A	120.1	C45—C46—C41	120.8 (3)
N2—C10—C9	124.1 (2)	C45—C46—H46	119.6
N2—C10—H10	118.0	O10—C47—O11	124.1 (2)
C9—C10—H10	118.0	O10—C47—C48	118.1 (2)
N3—C11—H11	118.4	O11—C47—C48	117.77 (19)
N3—C11—C12	123.28 (18)	C49—C48—C47	121.1 (2)
C12—C11—H11	118.4	C53—C48—C47	120.76 (19)
C11—C12—H12	120.0	C53—C48—C49	118.1 (2)
C11—C12—C13	120.09 (18)	O12—C49—C48	121.4 (2)
C13—C12—H12	120.0	O12—C49—C50	118.5 (2)
C12—C13—C14	116.58 (17)	C50—C49—C48	120.1 (2)
C12—C13—C18	121.10 (17)	C49—C50—H50	119.9
C14—C13—C18	122.32 (17)	C51—C50—C49	120.2 (2)
C13—C14—H14	120.1	C51—C50—H50	119.9
C15—C14—C13	119.89 (17)	C50—C51—H51	119.9
C15—C14—H14	120.1	C50—C51—C52	120.3 (2)
N3—C15—C14	123.20 (18)	C52—C51—H51	119.9
N3—C15—H15	118.4	C51—C52—H52	120.0
C14—C15—H15	118.4	C53—C52—C51	120.0 (2)
N4—C16—H16	118.3	C53—C52—H52	120.0
N4—C16—C17	123.43 (18)	C48—C53—H53	119.3
C17—C16—H16	118.3	C52—C53—C48	121.3 (2)
C16—C17—H17	120.1	C52—C53—H53	119.3
C16—C17—C18	119.76 (18)		
			
Co1—O1—C26—O2	1.3 (2)	C11—N3—C15—C14	1.1 (3)
Co1—O1—C26—C27	−178.48 (17)	C11—C12—C13—C14	0.4 (3)
Co1—O2—C26—O1	−1.3 (2)	C11—C12—C13—C18	−179.31 (19)
Co1—O2—C26—C27	178.48 (18)	C12—C13—C14—C15	−0.5 (3)
Co1—O4—C33—O5	98.2 (6)	C12—C13—C18—C17	−33.3 (3)
Co1—O4—C33—C34	−81.8 (6)	C12—C13—C18—C19	146.4 (2)
Co1^i^—O5—C33—O4	0.1 (2)	C13—C14—C15—N3	−0.2 (3)
Co1^i^—O5—C33—C34	−179.90 (12)	C13—C18—C19—C20	−179.36 (18)
Co1—N1—C1—C2	−178.47 (17)	C14—C13—C18—C17	147.0 (2)
Co1—N1—C5—C4	178.93 (16)	C14—C13—C18—C19	−33.2 (3)
Co1—N3—C11—C12	−178.99 (16)	C15—N3—C11—C12	−1.2 (3)
Co1—N3—C15—C14	178.79 (16)	C16—N4—C20—C19	−0.9 (3)
Co2—O7—C40—O8	3.61 (18)	C16—C17—C18—C13	179.20 (18)
Co2—O7—C40—C41	−176.61 (17)	C16—C17—C18—C19	−0.6 (3)
Co2—O8—C40—O7	−3.81 (19)	C17—C18—C19—C20	0.4 (3)
Co2—O8—C40—C41	176.41 (16)	C18—C13—C14—C15	179.13 (19)
Co2—O10—C47—O11	13.9 (3)	C18—C19—C20—N4	0.4 (3)
Co2—O10—C47—C48	−166.89 (13)	C20—N4—C16—C17	0.8 (3)
Co2—N4—C16—C17	179.12 (15)	C21—N5—C25—C24	−1.3 (3)
Co2—N4—C20—C19	−179.40 (16)	C21—C22—C23—C23^ii^	179.8 (3)
Co2—N5—C21—C22	−172.7 (2)	C21—C22—C23—C24	0.0 (4)
Co2—N5—C25—C24	172.38 (17)	C22—C23—C24—C25	−0.6 (3)
O1—C26—C27—C28	6.0 (3)	C23^ii^—C23—C24—C25	179.7 (2)
O1—C26—C27—C32	−173.9 (2)	C23—C24—C25—N5	1.3 (4)
O2—C26—C27—C28	−173.8 (2)	C25—N5—C21—C22	0.8 (4)
O2—C26—C27—C32	6.4 (3)	C26—C27—C28—O3	−2.1 (4)
O3—C28—C29—C30	−177.5 (3)	C26—C27—C28—C29	177.7 (3)
O4—C33—C34—C35	178.91 (18)	C26—C27—C32—C31	−179.6 (3)
O4—C33—C34—C39	1.4 (3)	C27—C28—C29—C30	2.7 (5)
O5—C33—C34—C35	−1.0 (3)	C28—C27—C32—C31	0.6 (4)
O5—C33—C34—C39	−178.55 (18)	C28—C29—C30—C31	−1.0 (6)
O6—C35—C36—C37	−179.0 (2)	C29—C30—C31—C32	−0.9 (6)
O7—C40—C41—C42	−2.6 (3)	C30—C31—C32—C27	1.1 (5)
O7—C40—C41—C46	178.6 (2)	C32—C27—C28—O3	177.8 (3)
O8—C40—C41—C42	177.2 (2)	C32—C27—C28—C29	−2.5 (4)
O8—C40—C41—C46	−1.7 (3)	C33—C34—C35—O6	1.8 (3)
O9—C42—C43—C44	179.5 (4)	C33—C34—C35—C36	−176.5 (2)
O10—C47—C48—C49	−175.88 (19)	C33—C34—C39—C38	177.4 (2)
O10—C47—C48—C53	4.6 (3)	C34—C35—C36—C37	−0.6 (4)
O11—C47—C48—C49	3.4 (3)	C35—C34—C39—C38	−0.2 (3)
O11—C47—C48—C53	−176.1 (2)	C35—C36—C37—C38	−0.6 (4)
O12—C49—C50—C51	179.4 (2)	C36—C37—C38—C39	1.3 (4)
N1—C1—C2—C3	−0.8 (3)	C37—C38—C39—C34	−0.9 (4)
N2—C6—C7—C8	0.0 (3)	C39—C34—C35—O6	179.3 (2)
N3—C11—C12—C13	0.6 (3)	C39—C34—C35—C36	1.0 (3)
N4—C16—C17—C18	0.0 (3)	C40—C41—C42—O9	1.0 (4)
N5—C21—C22—C23	−0.1 (5)	C40—C41—C42—C43	−178.2 (3)
C1—N1—C5—C4	−0.4 (3)	C40—C41—C46—C45	178.8 (3)
C1—C2—C3—C4	0.2 (3)	C41—C42—C43—C44	−1.3 (5)
C1—C2—C3—C8	179.9 (2)	C42—C41—C46—C45	−0.1 (4)
C2—C3—C4—C5	0.2 (3)	C42—C43—C44—C45	1.3 (7)
C2—C3—C8—C7	26.8 (3)	C43—C44—C45—C46	−0.8 (7)
C2—C3—C8—C9	−151.4 (2)	C44—C45—C46—C41	0.2 (5)
C3—C4—C5—N1	−0.1 (3)	C46—C41—C42—O9	179.9 (3)
C3—C8—C9—C10	176.7 (2)	C46—C41—C42—C43	0.7 (4)
C4—C3—C8—C7	−153.5 (2)	C47—C48—C49—O12	0.8 (3)
C4—C3—C8—C9	28.2 (3)	C47—C48—C49—C50	−179.83 (19)
C5—N1—C1—C2	0.8 (3)	C47—C48—C53—C52	179.7 (2)
C6—N2—C10—C9	1.4 (4)	C48—C49—C50—C51	0.1 (3)
C6—C7—C8—C3	−176.81 (19)	C49—C48—C53—C52	0.2 (3)
C6—C7—C8—C9	1.6 (3)	C49—C50—C51—C52	0.3 (4)
C7—C8—C9—C10	−1.6 (3)	C50—C51—C52—C53	−0.4 (4)
C8—C3—C4—C5	−179.44 (19)	C51—C52—C53—C48	0.2 (4)
C8—C9—C10—N2	0.1 (4)	C53—C48—C49—O12	−179.7 (2)
C10—N2—C6—C7	−1.5 (3)	C53—C48—C49—C50	−0.3 (3)

Symmetry codes: (i) −*x*+1, −*y*, −*z*+1; (ii) −*x*+1, −*y*+2, −*z*.

### Hydrogen-bond geometry (Å, º)

*Cg*4, *Cg*7 and *Cg*9 are the centroids of the N4/C16–C20, C34–C39 and C48–C53 rings, respectively.

**Table:**

*D*—H···*A*	*D*—H	H···*A*	*D*···*A*	*D*—H···*A*
O3—H3···O1	0.85 (3)	1.77 (3)	2.553 (3)	153 (4)
O6—H8···O5	0.84 (2)	1.86 (2)	2.587 (2)	145 (2)
O9—H13···O7	0.84 (2)	1.79 (2)	2.568 (3)	152 (4)
O12—H18···O11	0.85 (2)	1.74 (2)	2.531 (2)	154 (3)
O13—H26···O11	0.84 (1)	1.82 (2)	2.625 (2)	160 (2)
O13—H27···N2^i^	0.83 (2)	2.05 (2)	2.860 (3)	167 (2)
C25—H25···O8	0.93	2.55	3.143 (3)	122
C39—H39···O1	0.93	2.38	3.271 (3)	160
C5—H5···O12^iii^	0.93	2.59	3.324 (3)	136
C6—H6···O7^iv^	0.93	2.48	3.300 (3)	147
C12—H12···O3^v^	0.93	2.58	3.173 (3)	122
C15—H15···O6^vi^	0.93	2.51	3.292 (3)	142
C2—H2···*Cg*9^iv^	0.93	2.95	3.833 (2)	160
C31—H31···*Cg*4^vii^	0.93	2.85	3.681 (4)	149
C51—H51···*Cg*7^viii^	0.93	2.72	3.623 (3)	165

Symmetry codes: (i) −*x*+1, −*y*, −*z*+1; (iii) *x*, *y*−1, *z*+1; (iv) *x*+1, *y*−1, *z*+1; (v) −*x*+1, −*y*+1, −*z*+1; (vi) *x*−1, *y*, *z*; (vii) −*x*, −*y*+1, −*z*+1; (viii) *x*−1, *y*+1, *z*−1.
