# Supplementary material for: Crystal structure of a novel one-dimensional zigzag chain-like cobalt(II) coordination polymer constructed from 4,4′-bi­pyridine and 2-hy­droxy­benzoate ligands
Source: Acta Crystallogr E Crystallogr Commun. 2020 Jul 17;76(Pt 8):1302–6. doi: 10.1107/S2056989020009482 (PMC7405572; doi:10.1107/S2056989020009482)
Supplement: Supplementary file 3 [file e-76-01302-sup3.pdf]

Supporting Information

Crystal structure of a novel one-dimensional zigzag chain-like cobalt(II) coordination polymer constructed from mixed 4,4′-bipyridine and 2-hydroxybenzoate ligands

Thawanrat Saelim, Kittipong Chainok, Filip Kielar and Nanthawat Wannarit*

Figure Caption

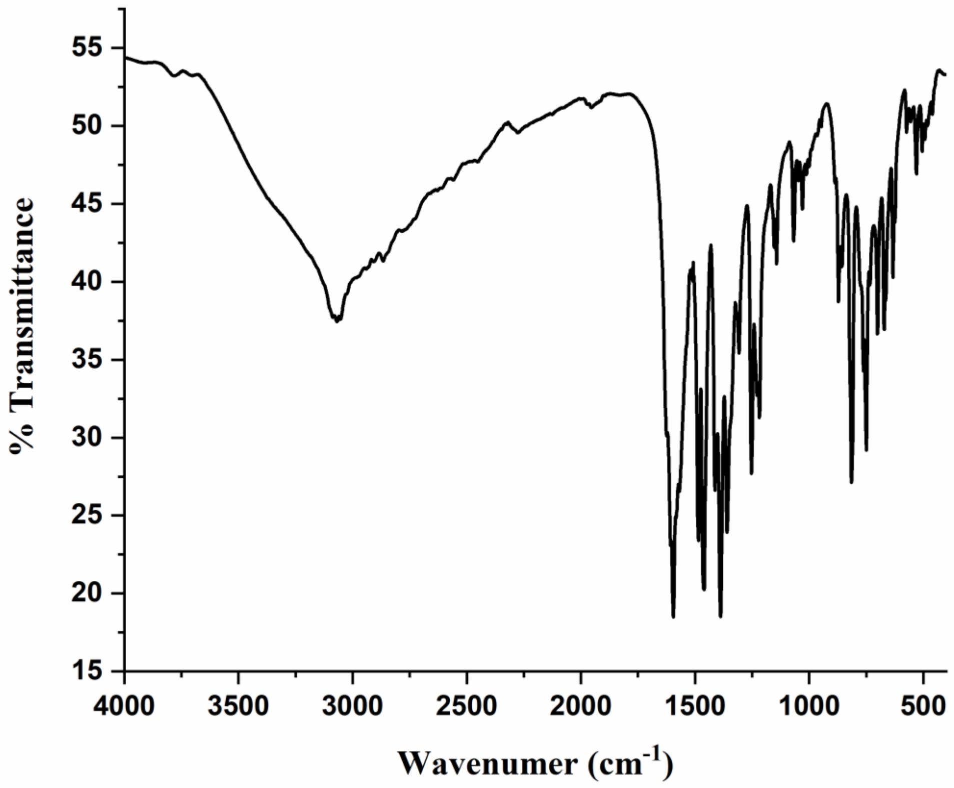

Figure S1

FT-IR spectrum of the title compound

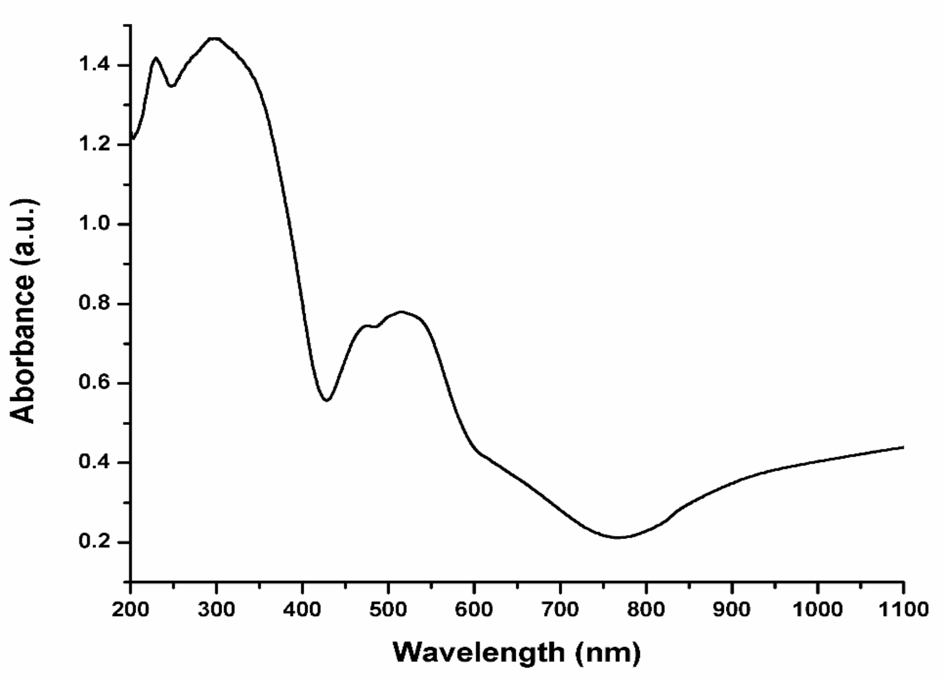

Figure S2

The solid-state diffuse reflectance spectrum of the title compound

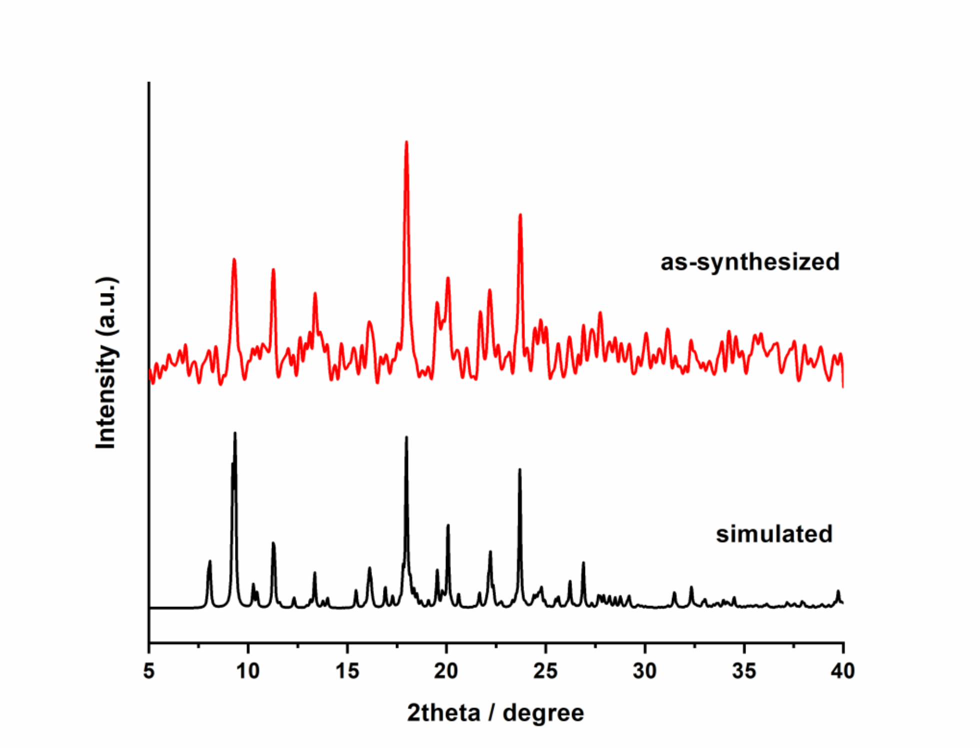

Figure S3

The powder XRD patterns of the title compound

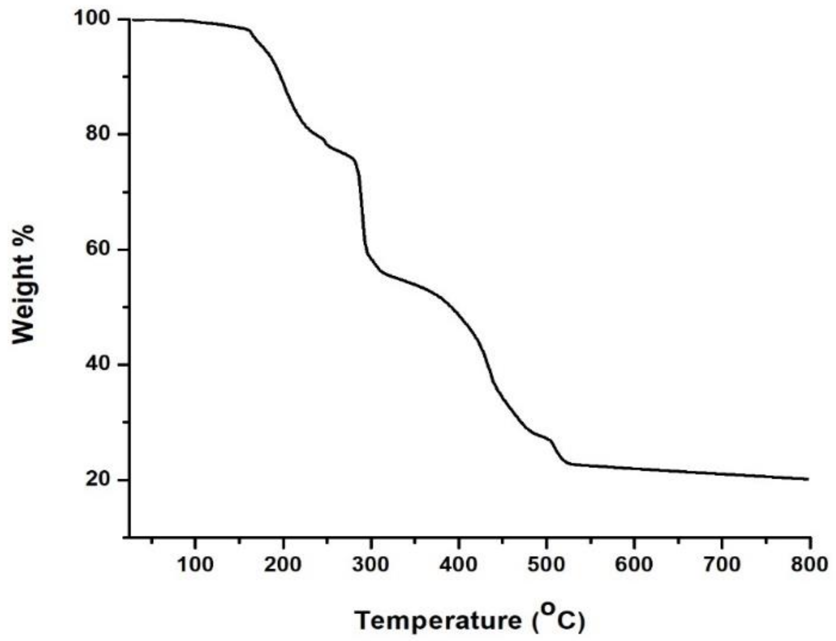

Figure S4

TGA curve of the title compound

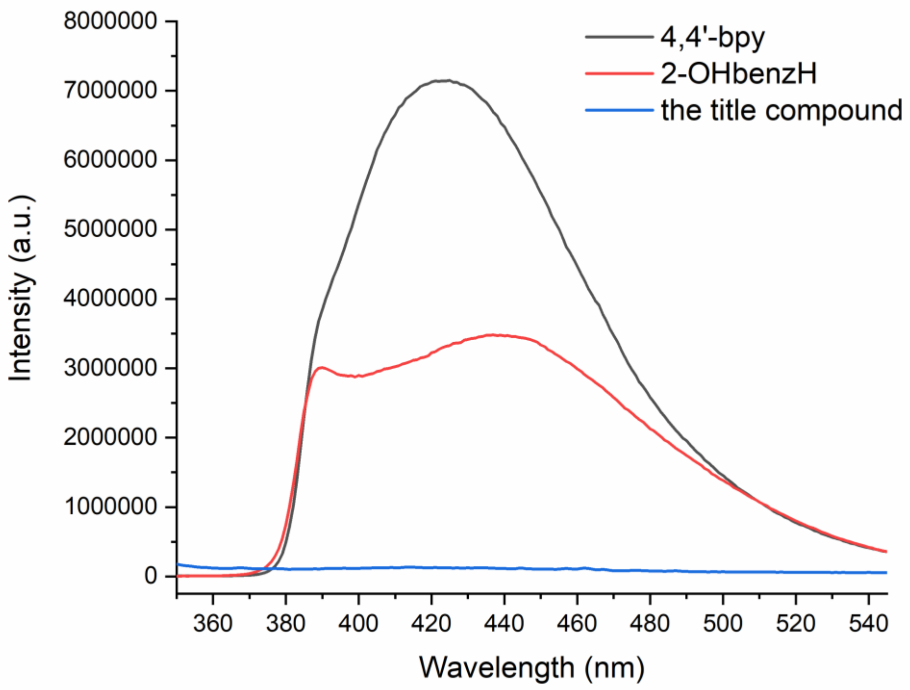

Figure S5

The solid-state PL emission spectra of the title compound and the free ligands at room temperature
